# Dissemination of hepatocellular carcinoma is mediated via chemokine receptor CXCR4

**DOI:** 10.1038/sj.bjc.6603251

**Published:** 2006-07-04

**Authors:** C C Schimanski, R Bahre, I Gockel, A Müller, K Frerichs, V Hörner, A Teufel, N Simiantonaki, S Biesterfeld, T Wehler, M Schuler, T Achenbach, T Junginger, P R Galle, M Moehler

**Affiliations:** 1 First Department of Internal Medicine, Johannes Gutenberg University of Mainz, Langenbeckstrasse 1, 55101 Mainz, Germany; 2 Department of Surgery, Johannes Gutenberg University of Mainz, Mainz, Germany; 3 Department of Pathology, Johannes Gutenberg University of Mainz, Mainz, Germany; 4 Third Department of Internal Medicine, Johannes Gutenberg University of Mainz, Mainz, Germany; 5 Department of Radiology, Johannes Gutenberg University of Mainz, Mainz, Germany

**Keywords:** CXCR4, chemokine, liver, hepatocellular, metastasis

## Abstract

In different tumour entities, expression of the chemokine receptor 4 (CXCR4) has been linked to tumour dissemination and poor prognosis. Therefore, we evaluated, if the expression of CXCR4 exerts similar effects in human hepatocellular carcinoma (HCC). Expression analysis and functional assays were performed *in vitro* to elucidate the impact of CXCL12 on human hepatoma cells lines. In addition, expression of CXCR4 was evaluated in 39 patients with HCC semiquantitatively and correlated with both, tumour and patients characteristics. Human HCC and hepatoma cell lines displayed variable intensities of CXCR4 expression. Loss of p53 function did not impact on CXCR4 expression. Exposure to CXCL12 mediated a perinuclear translocation of CXCR4 in Huh7/Hep3B cells and increased the invasive potential of Huh7 cells. In HCC patients, CXCR4 expression significantly correlated with progressed local tumours (T-status; *P*=0.006), lymphatic metastasis (N-status; *P*=0.005) and distant dissemination (M-status; *P*=0.009), as well as with a decreased 3-year-survival rate (*P*=0.01). In summary, strong expression of CXCR4 is significantly associated with progressed hepatocellular cancer.

Hepatocellular carcinoma (HCC) ranges among the most frequent cancer entities worldwide with an incidence of more than 500 000 cases per year and raising incidence rates in Western countries (Parkin *et al*, 2001). While mortality rates of many cancer entities are decreasing, hepatocellular cancer revealed an significant increase of death rates during the 1990s (Wingo *et al*, 2003). HCC is associated with liver cirrhosis commonly resulting from inflammatory liver disease, such as chronic hepatitis B (HBV), hepatitis C (HCV) and to a lesser extent from autoimmune-triggered hepatitis or cholangitis (AIH, PBC, PSC), but also from nonviral diseases, such as chronic alcohol intake or exposure to mycotoxin and aflatoxin B1 (Bosch *et al*, 1999). Hepatocarcinogenesis is considered a slow process in which diverse genomic alterations accumulate, altering the phenotype of hepatocytes and hence leading to multiple monoclonal and dysplastic hepatocyte populations (Thorgeirsson and Grisham, 2002). As progress of dysplastic hepatocytes to HCC might occur simultaneously in different foci or nodules, variable genomic alterations can be found within the same liver indicating a genetic heterogeneity of lesions (Feitelson *et al*, 2002). Molecular determinants including mutations and loss of heterozygosity (LOH) in certain tumour-suppressor genes (i.e. p53, IGF2R, RB1, PTEN) and oncogenes (i.e. N-ras, E2F4) have recently been proposed and summarised in the pathway of molecular pathogenesis of human HCC (Feitelson *et al*, 2002; Thorgeirsson and Grisham, 2002). Nonetheless, it seems very likely that additional pathogenic alterations instrumentally mediate the progression and dissemination of human HCC.

Tumour growth and metastatic dissemination are deemed to result from an intricate, dysregulated molecular machinery leading to diverse phenomena in tumour cells, such as resistance to the induction of apoptosis, immune escape mechanisms as well as invasion and migration capabilities. Recent data suggest that chemokine receptors may direct lymphatic and hematogenous spreading and may furthermore influence the sites of metastatic growth of different tumours (Arya *et al*, 2003).

Chemokines and their G-protein-coupled receptors were originally reported to mediate different pro- and anti-inflammatory responses (Zlotnik and Yoshie, 2000). The chemokine receptor 4 (CXCR4) was described to regulate the homing of lymphocytes in inflammatory tissues (Murdoch, 2000). Its natural ligand, CXCL12 (stromal cell-derived factor 1 alpha (SDF-1*α*)), is highly expressed by endothelial cells and in tissues of metastatic growth, such as lung, liver and lymph nodes, and attracts lymphocytes into these organs (Phillips *et al*, 2003). Increased CXCL12 production by biliary epithelial cells and proliferating bile ductules in diverse inflammatory liver diseases might play an additional role in the recruitment of CXCR4-positive inflammatory cells into the inflamed livers (Terada *et al*, 2003). Further data indicate that CXCR4 may be involved in the retention of alloactivated lymphocytes at sites of graft damage after liver transplantation (Goddard *et al*, 2001). Most recently, CXCR4 has shifted into focus as it might play an important role in tumour spreading.

High CXCR4 expression was associated with tumour dissemination in colorectal, breast and oral squamous cell carcinoma (Chen *et al*, 2003; Uchida *et al*, 2003; Schimanski *et al*, 2005). Supporting data from *in vitro* and murine *in vivo* tumour models underlined the key role of CXCR4 for tumour cell malignancy, as activation of CXCR4 induced migration, invasion and angiogenesis of cancer cells (Mori *et al*, 2004). Furthermore, downregulation of CXCR4 in melanoma cells led to decreased pulmonary metastasis in mice, both in number and size (Murakami *et al*, 2002).

However, no data are presently available on the expression of CXCR4 in human HCC and its impact on disease progression and prognosis. Therefore, we evaluated the expression of CXCR4 in hepatoma cell lines and HCC specimens and correlated the results with the patients' clinicopathological parameters and survival. Furthermore, we elucidated the impact CXCL12 exposure on CXCR4 localisation and on cellular proliferation and invasion, as well as of p53 function on CXCR4 expression.

## MATERIALS AND METHODS

### Cell culture

The human hepatoma cell lines Huh-7, Hep3B as well as CXCR4 deficient HepG2 and p53 dominant-negative transfected HepG2 were cultured in DMEM (Invitrogen, Germany) supplemented with 10% FCS, 100 units ml^−1^ penicillin, 100 *μ*g ml^−1^ streptomycin (Cambrex, Germany) and 1 mM L-glutamine (Invitrogen, Germany). P53 Dominant-negative transfected HepG2 were kindly provided by M Schuler (Third Departement of Internal Medicine, Johannes Gutenberg University, Mainz, Germany).

### Western blot analysis

Huh-7, Hep3B, HepG2 and p53 dominant-negative transfected HepG2 cells were washed with PBS and lysed in 0.5% NP-40 solution. Of protein 100 *μ*g was loaded on an 10% SDS–PAGE gel. The gel was transferred onto a PVDF membrane following seperation. The respective proteins were detected with anti-CXCR-4 (1 : 500, CIO115, Capralogics, USA; 1 : 1000 donkey anti-goat IgG 2nd antibody SC-2020 by Santa Cruz Biotechnology, CA, USA) and antiactin (1 : 1000, A2066, Sigma, Germany; 1 : 1000 goat anti-rabbit IgG 2nd antibody 170–6515 by Biorad, USA) and were visualised by ECL Western Blotting Analysis System (Amersham Biosciences, USA).

### Translocation assays

Translocation of CXCR4 under CXCL12 exposure was assayed in Huh-7, Hep3B and HepG2 cells. In brief, cells were seeded on chamber-slides and treated with CXCL12 (100 ng ml^−1^; Sigma, Germany) for 0, 2 or 24 h. IHC-CXCR4 staining and evaluation was performed as described below. To prove the observed effects, Huh7, Hep3B and HT29 cells were grown in chamber slides and exposed to CXCL12 for 0 or 2 h. After washing with PBS, the cells were fixed in methanol/acetone for 2 min. For immunofluorescence staining an anti-goat-FITC-conjugated IgG secondary antibody (F2016, Sigma, Germany) was used. Hoechst 33342 (Molecular Probes, Netherlands) was applied to stain nuclei. The cells were imaged directly in the chambers using a Zeiss LSM 510UV laser scanning microscope.

### FACS analysis

HT29 cells were grown in 12-wells in DMEM (Invitrogen, Germany) supplemented with 10% FCS, 100 units ml^−1^ penicillin, 100 *μ*g ml^−1^ streptomycin (Cambrex, Germany) and 1 mM L-glutamine (Invitrogen, Germany). One day before analysis FCS concentration was reduced to 1%. During analysis, cells were treated with CXCL12 for 0 or 2 h. After trypsination, cells were washed three times with PBS, finally solved in 25 *μ*l PBS and blocked with 25 *μ*l human fresh frozen plasma for 15 min at room temperature. Hereafter, 2 *μ*l of PE-conjugated anti-CXCR4 (Clone 12G5; BD Biosciences, Belgium) were added and incubated for 45 min at 37°C. Cells were again washed in PBS and finally analysed in a FACSCalibur (BD Biosciences; Belgium).

### Proliferation assays

Cells (Huh-7, Hep3B and HepG2 cells) (5 × 10^3^) were plated in 96-wells. After seeding, cells were either exposed to CXCL12 (Sigma, Germany) or the equivalent amount of solvent (0.1% BSA in PBS). The amount of cells per well was determined daily by luminescence assay according to the recommendations of the manufacturer (Celltiter-Glo, Cell Viability assay, Promega, USA). Each condition was performed in quadruplicates.

### Cellular migration/invasion assays

Invasion of Huh-7, Hep3B and HepG2 cells was assayed with the extracellular matrix covered QCM Chemotaxis 96-well cell invasion assay (8 *μ*M pore size; QCM Chemotaxis 96-well cell invasion assay kit; Chemicon International, USA). In brief, cells were serum starved for 24 h before initiation of the assay. Cells (4 × 10^4^) were resuspended in serum-free DMEM and added to the upper chamber. Consecutively, DMEM with 20% FCS and ±100 ng ml^−1^ CXCL12 was added to the lower chamber. Chambers were incubated for 24 h at 37°C in a humid atmosphere of 5% CO_2_. After incubation, the amount of invaded and migrated cells in the lower chamber was determined by fluorescence assay performed according to the recommendations of the manufacturer. Each condition was performed in quadruplicates.

### Tissue samples

Hepatocellular carcinoma tissue samples were obtained from 39 patients undergoing liver biopsy, hemihepatectomy or orthotopic liver transplantation for HCC at the University of Mainz. The morphological classification of the carcinomas was conducted according to World Health Organization (WHO) specifications. Patients were followed up on a regular basis depending on the procedure performed.

### Immunohistochemical staining

The avidin-biotin-complex method was used to detect the protein CXCR-4 (anti-CXCR-4, dilution 1 : 300; Capralogics, USA). Formalin-fixed and paraffin-embedded tissue were deparaffinised and subsequently microwaved in EDTA buffer. After preincubation with hydrogen peroxide, avidin/biotin blocking kit (Vector Laboratories Inc., USA) and rabbit serum (Vector Laboratories Inc., USA) the primary antibodies were applied for 1 h at room temperature. After incubation with the secondary antibody (rabbit anti-goat biotinylated; dilution 1 : 200, Vector Laboratories Inc., USA), the avidin-biotin-complex was added and the enzyme activity visualised with diaminobenzidine. Counterstaining was performed with haematoxylin. For negative controls only the secondary antibody was used. A negative control was performed for every HCC sample (*N*=39). For positive controls formalin-fixed and paraffin-embedded tissue samples of the human spleen were applied.

### Evaluation of immunostaining

Immunostaining was evaluated semiquantitatively by three authors independently (CCS, RB, NS), blinded to patient outcome and all clinicopathologic findings. The immunohistochemical staining was analysed according to a scoring method that we have previously validated and described (Schimanski *et al*, 2005): the tumours were classified into four groups based on the homogenous staining intensity: 0, absent; 1, weak; 2, intermediate; 3, strong staining. In the case of heterogeneous staining within the same sample, the respective, 0.5 points higher score was chosen, if more than 50% of cells revealed the higher staining intensity. If the evaluations did not agree the specimens were re-evaluated and then classified according to the assessment given most frequently by the observers.

### Statistics

The association of staining intensity with clinicopathological patterns was assessed with the *χ*^2^-test and with the unpaired Student's *t*-test, when appropriate. Survival rates were visualised applying the Kaplan–Meier curves, and *P*-values were determined by the log-rank test. *P*<0.05 was considered significant and *P*<0.001 highly significant in all statistical analyses.

## RESULTS

### CXCR4 expression in hepatoma cell lines

**Figure 1:**
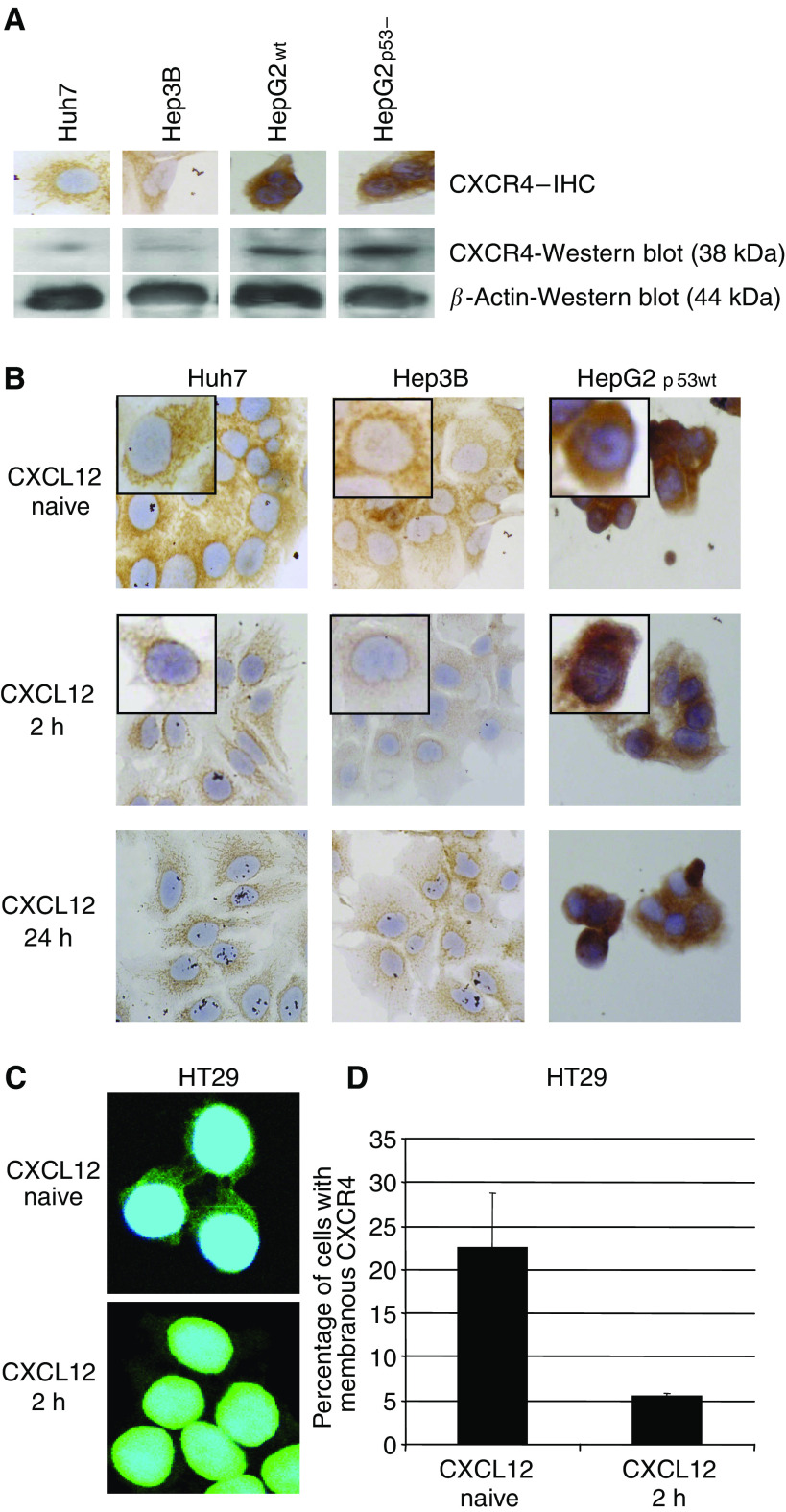
(**A**) Expression and immunohistochemical staining of CXCR4 in diverse human hepatoma cell lines. Huh7 and Hep3B cells revealed a weak CXCR4 expression, whereas HepG2 cell lines depicted medium CXCR4 staining, independent from p53 status. (**B**) Exposure to CXCL12 mediated a rapid perinuclear translocation of CXCR4 from the cytoplasma and membrane (inlet patch). This translocation was strongly evident in Huh7 and also in Hep3B cells, but absent in HepG2 cells. (**C**) Fusion of nuclear staining (blue) and CXCR4 staining (green). Exposure to CXCL12 mediated a rapid cytoplasmatic clearance and perinuclear translocation of CXCR4 in HT29. (**D**) FACS analysis revealed a significantly decreased amount of positive HT29 cells for membrane-bound-CXCR4 upon CXCL12 exposure.

CXCR4 expression analysis of human hepatoma cell lines revealed varying expression intensities as depicted by Figure 1A. CXCR4-immunostaining results correlated with the respective Western blot analysis (Figure 1A). CXCR4 immunostaining and Western blot analysis correlated with the respective immunofluorescence profile (confirmatory results not shown). Notably, p53 did not impact on CXCR4 expression.

### Translocation assays

CXCL12 induced translocation of CXCR4 from the membrane and cytoplasma to the perinuclear region was present in Huh7 and Hep3B cells, but absent in CXCR4-defecient HepG2 cell lines, which were used as a negative control and proof of principle in all functional assays (Figure 1B). Immunofluorescence analysis revealed, that exposure to CXCL12 mediated a rapid cytoplasmatic clearance and translocation of CXCR4 in Huh7 and Hep3B (confirmatory data not shown) and in HT29 (Figure 1C).

### FACS analysis

CXCL12 mediated a rapid decrease of HT29 cells positive for membrane-bound CXCR4 (−CXCL12: 22.46%; s.d. 6.24; +CXCL12: 5.49%; s.d. 0.38; *P*<0.01).

### Proliferation assays

**Figure 2:**
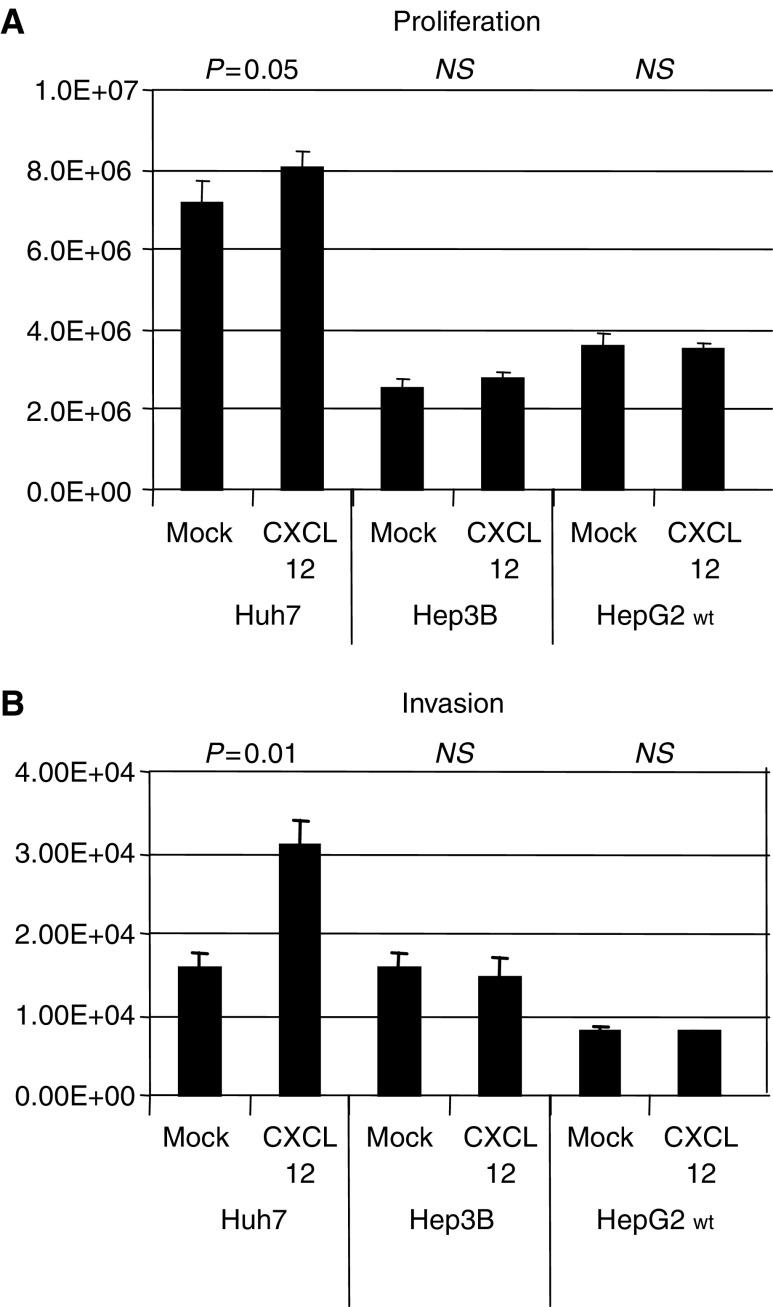
(**A** and **B**) Exposure to CXCL12-induced proliferation and invasion of Huh7, but not of Hep3B or HepG2 cells. While the impact of CXCL12 on invasion was highly significant, it was only marginally significant on proliferation.

The chemokine CXCL12 slightly stimulated proliferation of Huh-7 (Luminescence on day 4: 8037688±395512 IE *vs* 7146940±586960 IE; *P*=0.05), but not of Hep3B (Luminescence on day 4: 2745528±147741 IE *vs* 2533050±254525 IE; NS) or HepG2 (Luminescence on day 4: 3517047±173299 IE *vs* 3598328±294455 IE; NS) hepatoma cells (Figure 2A).

### Migration/invasion assays

The chemokine CXCL12 significantly stimulated migration of Huh-7 (Fluorescence: 30880±3298 IE *vs* 15705±1801 IE; *P*=0.001), but not of Hep3B (Fluorescence: 14367±2694 IE *vs* 15885±1559 IE; NS) or HepG2 (Fluorescence: 7608±110 IE *vs* 7956±416 IE; NS) hepatoma cells (Figure 2B).

### Tumour characteristics and patient profiles

The selected group of patients represent the typical characteristics of hepatocellular cancer in industrialised countries, except for a lower percentage of female patients and a prolonged survival resulting from hemihepatectomy and orthotopic liver transplantation for HCC. Patients characteristics are depicted in Table 1.

### Immunohistochemical staining of CXCR4 in HCC

**Figure 3:**
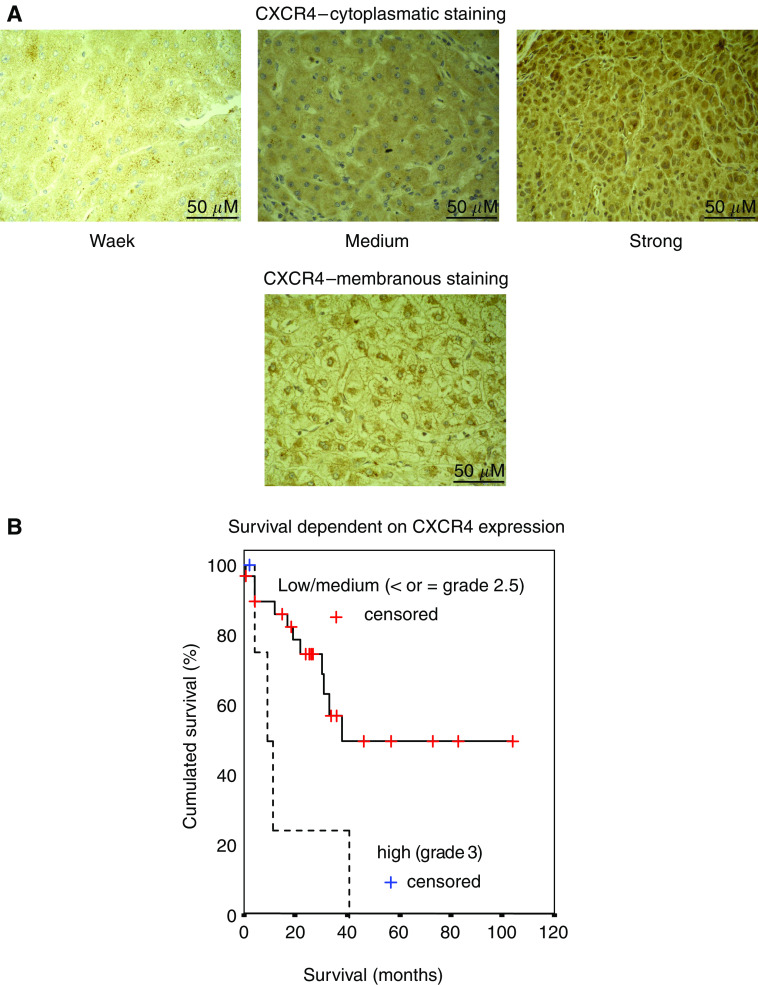
(**A**) Depicts the respective cytoplasmatic expression grades of CXCR4 (weak; medium and strong) as well as a rare sample of a membranous CXCR4 staining. (**B**) The probability of survival of HCC patients is given in relation to time after histological confirmation. Patients with grade 3 CXCR4 expression showed a significantly reduced 3-year-survival rate as compared to all other patients (*P*=0.01).

The staining for CXCR4 revealed predominantly a cytoplasmatic, and in few specimens an additional weak membranous or nuclear location of CXCR4 (Figures 3A–D). The respective expression rate for CXCR4 was 100% (39 out of 39) and varied from weak (8%), intermediate (56%) to strong (36%).

Negative controls of human hepatocellular cancer remained negative for every tissue sample (*N*=39, not shown). Splenic lymphocytes revealed a strong CXCR4 expression matching human hepatocellular cancer tissue or cell lines with strong expression of CXCR4. Similarly, inflammatory infiltates of the liver depicted a strong CXCR4 expression.

### Relevance of CXCR4 expression in HCC

Strong CXCR4 expression did correlate with local progression and proliferation of the primary tumour as indicated by the T-status (TNM classification; *P*=0.006; Table 2), with lymph node involvement (N-status*; P*=0.005) and with distant metastases (M-status; *P*=0.009; Table 2). Evidently, CXCR4 expression did not have impact on the grading, medium age or gender. Concerning survival, a strong CXCR4 expression (grades 2.5 and 3) could not be significantly correlated with survival (*P*=0.2). Noteworthy, if a subgroup with grade 3 expression (homogenous strong expression) was compared to the rest (heterogenous strong or lower) a decreased 3-year-survival rate of 25%, as compared to 57% was observed. This difference is depicted in the respective survival plot (Figure 3B, log rank *P*=0.01).

## DISCUSSION

So far, no data have been available on the expression of CXCR4 in human HCC and its impact on disease progression and prognosis. In other tumour entities, expression of the chemokine receptor CXCR4 has been linked to tumour dissemination and poor prognosis. Therefore, we analysed the expression profile of CXCR4 in a series of human hepatoma cell lines and HCC patients. Diverse external factors, such as hypoxia (hif-1-pathway) and the activation of adenosine receptors, as well as internal cellular alterations like the inactivation of tumour-suppressor genes pVHL, p53 or the overexpression of NFkB have been defined as important molecular regulators of the CXCR4 expression (Helbig *et al*, 2003; Staller *et al*, 2003; Mehata *et al*, 2004). To verify the regulation of CXCR4 by p53 in human HCC, we investigated the CXCR4 expression profile in p53 wt and dominant-negative transfected HepG2 cells, Huh7 and Hep3B cells (Figure 1A and B). The human hepatoma cell lines analysed revealed different intensities of a predominantly cytoplasmatic CXCR4 expression. Interestingly, the p53 dominant-negative HepG2 cells revealed unchanged levels of CXCR4 expression as compared to wt HepG2 cells, failing to confirm a regulatory mechanism of p53 on CXCR4 expression in transformed hepatic cells, although a p53-mediated impact on CXCR4 expression has been previously proposed for other cell lines (Mehata *et al*, 2004). Exposure to CXCL12, the ligand of CXCR4, mediated a rapid perinuclear translocation of CXCR4 in Huh7 and Hep3B, but not in HepG2 cells within two hours, indicating a cellular activation with pseudopodia formation as classically observed in epithelial mesenchymal transition (EMT). A clathrin-mediated endocytosis following activation and perinuclear translocation of CXCR4 to the perinuclear rab4-, rab5- and rab11-compartment of receptor recycling has previously been reported in human CD34+ haematopoetic progenitor cells (Zhang *et al*, 2004). Unfortunatelly, FACS analysis of CXCR4 in Huh7 and Hep3B cells previously failed for technical reasons, due to a weak expression level of CXCR4; HepG2 cells could also not be applied due to the defect receptor. In order to confirm the observed translocation in human carcinoma cells we used HT29 cells, which had previously revealed a strong CXCR4 expression and an intact CXCR4 receptor (Schimanski *et al*, 2005). FACS and immunofluorescence analysis confirmed an internalisation of CXCR4 and a rapid perinuclear translocation upon CXCL12 exposure. In hepatoma cells, exposure to CXCL12 increased proliferation and invasion of Huh7, but not of Hep3B or HepG2 cells. While the impact of CXCL12 on proliferation of Huh7 cells was only marignal, it was significant on invasion. The observed absence of CXCR4 translocation and CXCL12 induced proliferation/invasion in HepG2 cells, confirms earlier data, indicating a loss of chemokine CXCL12-mediated CXCR4 signalling and receptor internalisation in HepG2 cells due to a receptor defect (Mitra *et al*, 2001). In contrast to HepG2 cells, a defect of the intracellular signal cascade has to be proposed for Hep3B cells, resulting in a resistance to CXCL12-mediated phenomena in spite of receptor translocation. Taken together, our data are in line with *in vitro* results from other tumour entities, revealing that CXCR4 is essential for proliferation, adhesion, migration and invasion of CXCR4 expressing cancer cells, although the impact of CXCL12 in Huh7 was dramatically stronger on invasion than on proliferation (Mori *et al*, 2004; Schimanski *et al*, 2005).

The liver, lungs and lymph nodes are major target organs for metastases of diverse cancers in men. Recent publications on CXCR4 are in line with the ‘homing’ theory as CXCR4 expression comediates dissemination of primary tumours to different organs through the chemotactic factors CXCL12. Homing factors, influencing chemotaxis to target organs, have been proposed as the filter function of these organs not solely explains the growth of metastases (Stetler-Stevenson and Kleiner, 2001). Herein, chemokine CXCL12 expression seems to be most intense in typical ‘homing organs’ such as lungs, bone marrow, liver and lymph nodes as compared with other nonhoming tissues (Phillips *et al*, 2003). Furthermore, endothelial cells, such as pulmonary endothelial cells have been shown to coexpress CXCL12 and vascular cellular adhesion molecule (VCAM)-1, thus mediating tumour-cell/endothelial-cell attachment (Cardones *et al*, 2003). In particular, binding of CXCL12 on CXCR4-induced *β*-integrin expression on cancer cells, which was critical to adhesion of cancer cells to VCAM on endothelial cells (Burger *et al*, 2003). In a murine model only CXCR4 expressing, but not CXCR4-deficient CT-26 colon carcinoma cells grew into macrometastases; nonetheless both cell types were able to colonise livers and lungs after injection (Zeelenberg *et al*, 2003). Interestingly, oval cells, known to express CXCR4 as well, have been reported to migrate to the liver along a CXCL12 gradient, established by injured hepatocytes, inducing oval-cell-aided liver regeneration in hepatitis (Hatch *et al*, 2002; Mavier *et al*, 2004). Thus, hepatic tumour dissemination and hepatic regeneration share common pathways of chemotaxis.

In our experiments, immunohistochemical staining of human HCC specimens displayed cytoplasmatic CXCR4 expression with variable intensities, matching the observations made in human colorectal cancer cell lines. A membranous or nuclear localisation of CXCR4 was observed in fewer cases, but an inducible translocation of CXCR4 from the cytoplasma to the membrane has been reported previously (Richard *et al*, 2004).

In our patients, a strong CXCR4 expression was significantly associated with locally progressed tumours, lymph node and distant metastases. In contrast to other cancer entities, progressed T-status of HCC is defined as intrahepatic growth and spread of the respective hepatocellular cancer. Thus, our results not necessarily imply a substantial influence of CXCR4 on the proliferation, but certainly on intrahepatic, lymphatic and hematogenous dissemination of HCC.

Therefore, our results are in line with recent publications from our group and others, reporting a similar effect of CXCR4 on disease dissemination in other tumour entities, such as non-small-cell lung cancer (NSCLC), colorectal or breast cancer (Schimanski *et al*, 2005). Furthermore, CXCR4 expression was found upregulated in glioblastoma and its consecutive receptor inhibition was followed by tumour cell arrest (Rubin *et al*, 2003). In osteosarcoma, the level of CXCR4 expression was inversely correlated with overall survival, but positively associated with detection of metastasis (Larverdiere *et al*, 2004). Likewise, a grade 3 CXCR4 expression in our HCC patients was associated with a significantly reduced 3-year-survival, due to hepatic failure following diffuse intrahepatic HCC dissemination. The prolonged average survival rate of our patients as compared to reported survival rates in standard HCC patients resulted from a high rate of orthotopic liver transplantations in our patient group. Nevertheless, the impact of a strong CXCR4 expression on disease progression might depend on the type and location of the primary carcinoma and most likely on other parameters, which have not been defined yet. In line with this theory, a strong CXCR4 expression was associated with noninvasive gastric cancers and not with lymph node metastases (Kwak *et al*, 2004).

## CONCLUSION

Certain chemokines have been proposed to distinctly contribute to tumour growth, dissemination and local immune scape (Balkwill and Mantovani, 2001). Our *in vitro* and *in vivo* results are in line with these data for human HCC. Strong expression of CXCR4 by HCC was significantly associated with intrahepatic, nodal and distant dissemination. Thus, CXCR4 apparently plays a relevant role during HCC progression. Further efforts will be necessary to evaluate the inhibition of dissemination by CXCR4 antagonists.

## Figures and Tables

**Table 1:** Patient and tumour characteristics

	**Patient characteristics**
Total number	39
	
Median age (years)	60.6
	
*Gender*	
Female	4 (10%)
Male	35 (90%)
	
*T-status*	
1	8 (20%)
2	10 (26%)
3	16 (41%)
4	5 (13%)
	
*N-status*	
0	24 (62%)
1	10 (26%)
2	4 (10%)
Unknown	1 (2%)
	
*M-status*	
0	26 (67%)
1	12 (31%)
Unknown	1 (2%)
	
*Grading*	
1	3 (8%)
2	25 (64%)
3	11 (28%)
	
3-year-survival	54%

**Table 2:** Patient and tumour characteristics dependent on intensity of CXCR4 expression

	**CXCR4 expression**			
	**Weak (1)**	**Intermediate (2)**	**Strong (3)**	**Statistics**
Total number	3 (8%)	22 (56%)	14 (36%)	
				
Average age (years)	61.2		60.4	NS
				
*Gender*				
Female	2		2	NS
Male	23		12	
				
*T-status*				
1+2	16		2	*P*=0.006
3+4	9		12	
				
*N-status* a				
0	20		4	*P*=0.005
+	5		9	
				
*M-status* a				
0	21		5	*P*=0.009
+	4		8	
				
*Grading*				
1+2	17		11	NS
3+4	8		3	
				
3-year-survival	53%		55%	NS
	58%		25%	*P*=0.01

a N and M status could not be obtained from one patient.

NS=not significant.
